# Cold‐inducible protein RBM3 mediates hypothermic neuroprotection against neurotoxin rotenone via inhibition on MAPK signalling

**DOI:** 10.1111/jcmm.14588

**Published:** 2019-08-22

**Authors:** Hai‐Jie Yang, Rui‐Juan Zhuang, Yuan‐Bo Li, Tian Li, Xin Yuan, Bing‐Bing Lei, Yun‐Fei Xie, Mian Wang

**Affiliations:** ^1^ School of Life Science and Technology Xinxiang Medical University Xinxiang China; ^2^ Henan Key Lab of Biological Psychiatry Second Affiliated Hospital of Xinxiang Medical University Xinxiang China; ^3^ School of Pharmaceutical Sciences Xiamen University Xiamen China

**Keywords:** hypothermia, MAPK signalling, Parkinson's disease, RBM3, rotenone

## Abstract

Mild hypothermia and its key product, cold‐inducible protein RBM3, possess robust neuroprotective effects against various neurotoxins. However, we previously showed that mild hypothermia fails to attenuate the neurotoxicity from MPP^+^, one of typical neurotoxins related to the increasing risk of Parkinson disease (PD). To better understand the role of mild hypothermia and RBM3 in PD progression, another known PD‐related neurotoxin, rotenone (ROT) was utilized in this study. Using immunoblotting, cell viability assays and TUNEL staining, we revealed that mild hypothermia (32°C) significantly reduced the apoptosis induced by ROT in human neuroblastoma SH‐SY5Y cells, when compared to normothermia (37°C). Meanwhile, the overexpression of RBM3 in SH‐SY5Y cells mimicked the neuroprotective effects of mild hypothermia on ROT‐induced cytotoxicity. Upon ROT stimulation, MAPK signalling like p38, JNK and ERK, and AMPK and GSK‐3β signalling were activated. When RBM3 was overexpressed, only the activation of p38, JNK and ERK signalling was inhibited, leaving AMPK and GSK‐3β signalling unaffected. Similarly, mild hypothermia also inhibited the activation of MAPKs induced by ROT. Lastly, it was demonstrated that the MAPK (especially p38 and ERK) inhibition by their individual inhibitors significantly decreased the neurotoxicity of ROT in SH‐SY5Y cells. In conclusion, these data demonstrate that RBM3 mediates mild hypothermia‐related neuroprotection against ROT by inhibiting the MAPK signalling of p38, JNK and ERK.

## 1 INTRODUCTION

Parkinson's disease (PD) is a complex multifactorial disorder that is primarily characterized by the loss of nigral dopaminergic neurons.^1^, ^2^, ^3^ Although the aetiology and pathogenesis of PD are poorly understood, environmental exposure‐induced mitochondrial dysfunction is frequently observed in the substantia nigra and cortex in PD.^4^ Rotenone (ROT) is a common pesticide and a known inhibitor of mitochondrial complex I. It can lead to relatively specific damage to dopaminergic neurons and increase the risk of PD by 2‐3 folds on average.^5^, ^6^, ^7^ Upon binding to mitochondrial complex I, ROT can increase oxidative stress and induce apoptosis in neurons and neuroblastoma cells.^8^ Therefore, ROT‐induced neuronal death is often used as an in vitro model for PD studies.

Cooling is an efficient tool to ameliorate the side‐effects of brain surgery.^9^ In the clinic, mild hypothermia (32‐35°C) has been widely used to improve outcome of acute brain injuries, including hypoxic‐ischaemic encephalopathy in newborn infants and cardiac arrest in adults.^9^ Hypothermic neuroprotection may be associated with the inhibition of intracellular signalling, oxidative stress, excitotoxicity, inflammation and apoptotic cell death.^10^, ^11^, ^12^ Recently, mild hypothermia was also found to confer neuroprotective effects in neurodegenerative diseases, based on studies of Alzheimer‐type and prion‐infected mouse models.^13^ However, we recently found that mild hypothermia does not provide neuroprotection against MPP^+^, another known neurotoxin related to PD.^14^ To better understand the influence of hypothermia on PD progression, more PD cell models, such as ROT‐based one should be included.

The cold‐inducible protein RBM3 (RNA‐binding motif protein 3) is the first protein to be synthesized in response to hypothermia in mammalian cells.^15^ It is an RNA‐binding protein with 157 amino acids, belonging to a small group of proteins whose expression increases with cooling, while the expression of most other proteins decreases.^16^ There is growing evidence indicating that RBM3 is a crucial factor mediating hypothermia‐induced neuroprotective effects. In primary neurons and cortical organotypic slice cultures, mild hypothermia markedly increased RBM3 expression and rescued neuronal cells from forced apoptosis.^17^, ^18^, ^19^ In Alzheimer‐type and prion‐infected mouse models, RBM3 was identified as a crucial mediator of hypothermia‐induced neuroprotection,^13^ revealing that RBM3 induction/overexpression may provide protection not only in cases of acute brain injury but also in neurodegenerative diseases. We previously showed that the overexpression of RBM3 protects dopaminergic neuroblastoma cells SH‐SY5Y from the apoptosis induced by nitric oxide (NO), ultra‐violet (UV) irradiation and an overdose of retinoic acid (RA), whereas RBM3 knockdown revokes the neuroprotective effects of mild hypothermia in SH‐SY5Y cells.^20^, ^21^, ^22^ In MPP^+^‐based PD cell model, RBM3 also provides a robust protection, although mild hypothermia does not.^14^ However, it is undetermined whether RBM3 provides such neuroprotective effects in ROT‐based PD cell model.

In this study, we hypothesized that mild hypothermia protects human neuroblastoma SH‐SY5Y cells from ROT‐induced neurotoxicity (in vitro model of PD) via the action of RBM3. To test our hypothesis, we evaluated the ability of mild hypothermia to prevent apoptotic cell death in SH‐SY5Y cells and tested if RBM3 overexpression could recapitulate the same neuroprotective effects. We investigated the influence of RBM3 and mild hypothermia on p38, JNK and ERK MAPK signalling pathways. We also tested if the inhibition of MAPK signalling pathways could rescue ROT‐induced apoptosis.

## 2 MATERIALS AND METHODS

### 2.1 Cell culture, hypothermia and drug treatment

The human neuroblastoma cell line SH‐SY5Y (passage ≤ 25) was maintained in Dulbecco's modified Eagle's medium (DMEM) supplemented with 10% foetal bovine serum (FBS), 100 U/mL penicillin and 100 μg/mL streptomycin, at 37°C and 5% CO_2_ in a fully humidified incubator. The exposure to mild hypothermia was a 1‐d incubation at 32°C, while the control cells for the same experiment were simultaneously exposed to normothermia at 37°C.

Rotenone (ROT) was purchased from Sigma‐Aldrich and dissolved in dimethyl sulfoxide (DMSO). SH‐SY5Y cells were treated with a concentration of 0.5 μmol/L ROT for durations that are specified for each method and in the figure legends. ROT was made fresh prior to each treatment, and all treatments were one‐time, single‐dose exposures. The p38‐specific inhibitor (SB203580; Sigma‐Aldrich) and MEK inhibitor (U0126; Cell Signaling Technologies) were used at a 10 μmol/L concentration, whereas the JNK inhibitor (SP600125; Sigma‐Aldrich) was used at 25 μmol/L. The SH‐SY5Y cells were incubated with inhibitors or DMSO (vehicle control) for 1 hour prior to further treatment such as ROT treatment.

### 2.2 RBM3 overexpression

The sequence of the human *Rbm3* gene (GenBank NM_006743.4) was optimized for enhanced mammalian expression, chemically resynthesized by Sangon, and cloned into the expression vector pXJ40‐myc between *Bam*H I and *Xho* I sites. The SH‐SY5Y cells were transfected either with the RBM3‐encoding plasmid “pXJ40‐myc‐RBM3” or the empty vector “pXJ40‐myc” as a control using the Lipofectamine 3000 transfection reagent (Invitrogen) according to the manufacturer's instructions. At 24 hours post‐transfection, cells were used for further experiments. The RBM3 protein expressed from the pXJ40 vector included a myc tag and could therefore be differentiated from the endogenous RBM3 by molecular weight.

### 2.3 Cell viability assay

The 3‐(4, 5‐dimethythiazol‐2‐yl)‐2,5‐diphenyl tetrazolium bromide (MTT) was used to assess cell viability as described in our previous study.^21^ Briefly, SH‐SY5Y cells (1.0 × 10^4^/well) were seeded in a 96‐well plate and incubated overnight, followed by treatment with ROT as defined in the figure legend. To determine cell viability, 1 mg/mL MTT solution was added to the culture and incubated for 4 hours at 37°C. Then, the culture medium was removed and 150 μL DMSO was added to dissolve the purple formazan crystals, which are only formed in living cells. The colorimetric measurement was taken at an absorbance of 490 nm using a microplate reader (Molecular Devices).

### 2.4 Western blotting

Cells were harvested after treatment with mild hypothermia or ROT (0.5 µmol/L) and washed twice with cold phosphate‐buffered saline (PBS) (3.2 mmol/L Na_2_HPO_4_, 0.5 mmol/L KH_2_PO_4_, 1.3 mmol/L KCl, 140 mmol/L NaCl, pH 7.4). Then, they were treated with cold lysis buffer (20 mmol/L Tris pH 7.5, 150 mmol/L NaCl, 1 mmol/L EDTA, 1 mmol/L EGTA, 5 mmol/L NaF, 0.5% Triton X‐100, 2.5 mmol/L sodium pyrophosphate, 1 mmol/L β‐glycerolphosphate, 1 mmol/L Na_3_VO_4_, 1 mg/L leupeptin and 0.5% Na‐deoxycholate) followed by centrifugation at 12 000 *g* for 15 minutes at 4°C. The supernatant was collected, and protein concentration was determined by Bradford assay (Bio‐Rad). For Western blotting, proteins were separated by electrophoresis on an 8%‐15% SDS‐PAGE gel and transferred to a PVDF membrane (Millipore). After blocking with TBST (Tris‐buffered saline with 0.1% Tween‐20) containing 5% skim milk for 45 minutes, the membranes were incubated with the desired primary antibody for 1 hour at room temperature. Primary antibodies used were for p38 (#9212), phosphorylated (p‐) p38 (#9211), p‐ERK1/2 (#4370), p‐JNK1/2(#4668), AMPK (#4150), p‐AMPK (#2535), p‐GSK3β (#9331), IκBα (#4814), p‐IκBα (#2859), cleaved PARP (#9541), Bcl‐2 (#2870), Bax (#5023) and β‐actin (#4970) from Cell Signaling Technology (Beverly); anti‐JNK1 (sc‐474) and anti‐ERK2 (sc‐154) from Santa Cruz; anti‐GSK3β (#D160468) from BBI Life Sciences, antibodies against RBM3 (ab134946) from Abcam. After washing with TBST three times, the membranes were further incubated with horseradish peroxidase–conjugated secondary antibodies (Vazyme Biotech) for 1 hour at room temperature and developed with Pierce's West Pico Chemiluminescence substrate. The immunoreactive bands were visualized by the Luminescent image analyser (Amersham Imager 600, GE Healthcare). To confirm equal protein loading, the membranes were stripped (2% SDS, 100 mmol/L Tris pH 6.8) and immunoblotted for β‐actin.^23^ The protein band density was measured by the ImageJ 1.50 software (NIH). The band density for proteins exhibiting a double‐banded pattern was quantified as the sum of both individual band densities, or the relevant band is indicated by an arrow in the figure. Phosphorylated protein levels were quantified as a relative density of phospho/total protein, while all other proteins were quantified as a relative density of protein/β‐actin density.

### 2.5 TUNEL and DAPI staining

After ROT treatment (0.5 µmol/L for 24 hours), cell apoptosis was detected with the one‐step TUNEL kit as per the manufacturer's instructions (Beyotime Biotechnology). Briefly, the cells were fixed in 4% paraformaldehyde for 15 minutes and permeabilized with 0.1% Triton X‐100 for 5 minutes. After several washes with PBS, 50 µL of TUNEL reaction mixture was added to the cells, and they were incubated at 37°C for 1 hour in the dark. Finally, the cells were examined under a fluorescent light microscope (Leica). For DAPI staining, the SH‐SY5Y cells were seeded in 35‐mm plates. After treatment, the cells were washed in cold PBS and fixed with 4% paraformaldehyde for 30 minutes. After three more washes in cold PBS, cells were incubated with a 5 μg/mL 4,6‐diamidino‐2‐phenylindole dihydrochloride (DAPI) (Sangon Biotech) solution for 20 minutes. Finally, the cells were again washed in cold PBS three times and examined using the same fluorescent light microscope to visualize the DAPI‐stained cell nuclei.

### 2.6 Statistical analysis

All the experiments were repeated three times. Data were analysed by Student's *t* test and expressed as mean ± standard deviation (SD). *P* value <0.05 was statistically considered significant.

## 3 RESULTS

### 3.1 Mild hypothermia prevents rot‐induced neurotoxicity in SH‐SY5Y cells

**Figure 1:**
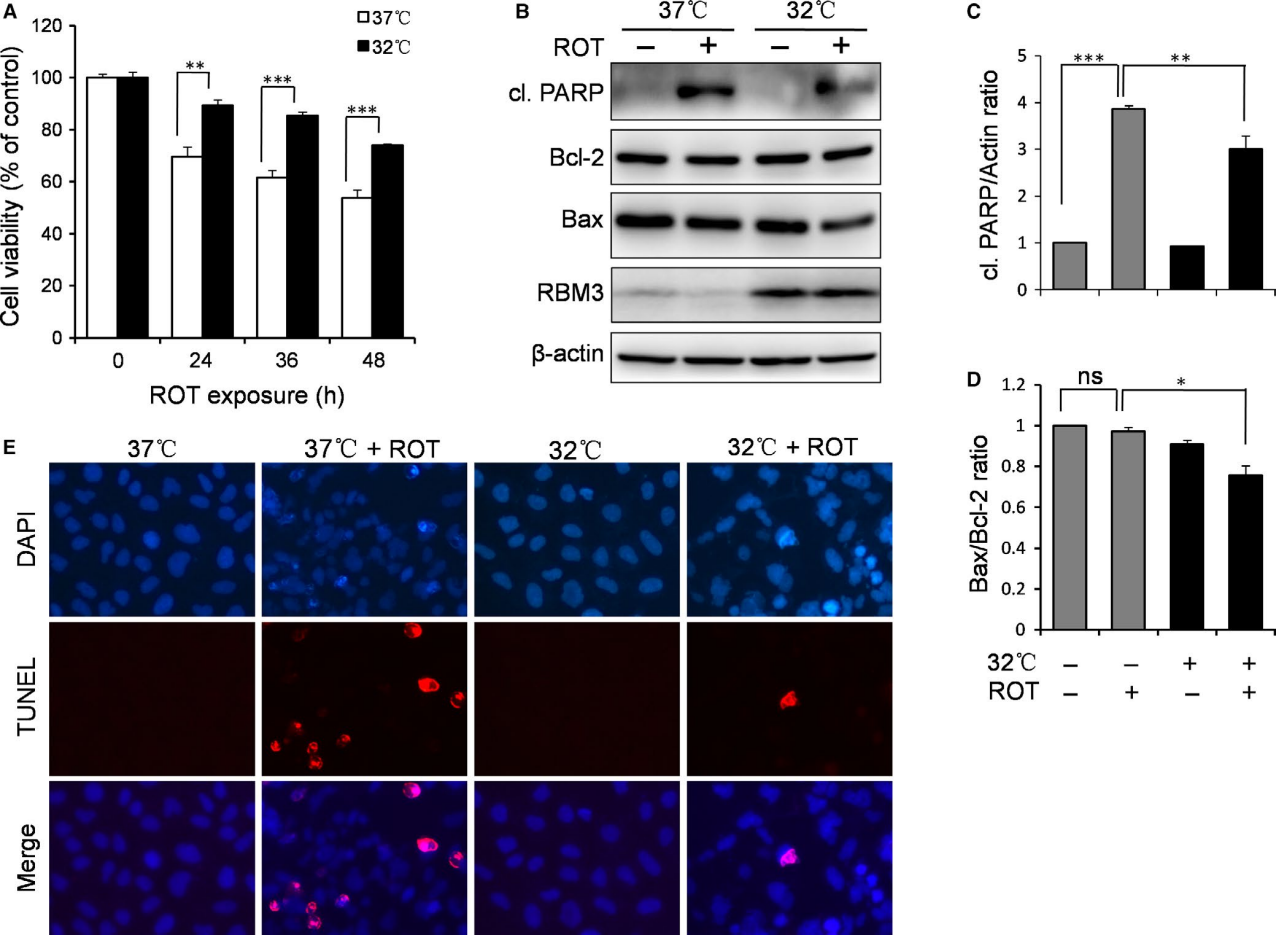
Mild hypothermia protects against ROT‐induced apoptosis in SH‐SY5Y neuroblastoma cells. Cells were pre‐cultured under normothermic (37°C) or mild hypothermic (32°C) conditions for 1 d and treated with 0.5 μmol/L ROT. A, An MTT assay was performed to examine the effects of mild hypothermia on cytotoxicity after ROT treatment for a time course of 0, 24, 36 and 48 h. B, Cells were treated with ROT for 24 h, after which Western blotting of cleaved (cl.) PARP, Bcl‐2, Bax, RBM3 and β‐actin was performed. C, D, The levels of cl. PARP and Bcl‐2 were quantified by densitometry and normalized to β‐actin and Bax, respectively. E, TUNEL staining after 24 h of ROT treatment evaluated the protective effects of mild hypothermia on ROT‐induced apoptosis. All data are representative of three independent experiments, and values are mean ± SD.; **P < 0.01 and ***P < 0.001 vs normothermia pre‐cultured cells

Human neuroblastoma SH‐SY5Y cells were used to evaluate the effects of mild hypothermia on ROT‐induced neurotoxicity. As shown in Figure S1, 0.5 μmol/L ROT produces significant cytotoxicity in SH‐SY5Y cells and is thus the concentration that we used for all experiments in our study. Cells were exposed to hypothermia (32°C) or normothermia (37°C) for 1 d and then subjected to ROT treatment. The MTT assay for cell viability showed that mild hypothermia significantly reduced the cytotoxicity of ROT towards SH‐SY5Y cells at all time‐points compared to normothermia (Figure 1A). In line with this, ROT‐treated cells exposed to hypothermia showed a markedly reduced expression of apoptosis‐related protein, cleaved poly (ADP‐ribose) polymerase (PARP), compared to the control cells exposed to normothermia (*P* = 0.0075; Figure 1B,C). This suggests that mild hypothermia suppresses ROT‐induced apoptosis. In response to ROT exposure, the apoptosis‐related protein Bax was also down‐regulated in hypothermia pre‐treated cells, whereas no alteration of Bcl‐2 was seen (Figure 1B,D). More convincingly, we observed less TUNEL staining in cells exposed to hypothermia compared to normothermia, showing that hypothermic pre‐treatment attenuates ROT‐induced apoptosis in SH‐SY5Y cells (Figure 1E). Taken together, the data from the MTT assay, Western blotting and TUNEL staining strongly suggest that mild hypothermia attenuates ROT‐induced neurotoxicity in SH‐SY5Y cells.

### 3.2 Overexpression of rbm3 protects SH‐SY5Y cells from rot‐induced apoptosis

**Figure 2:**
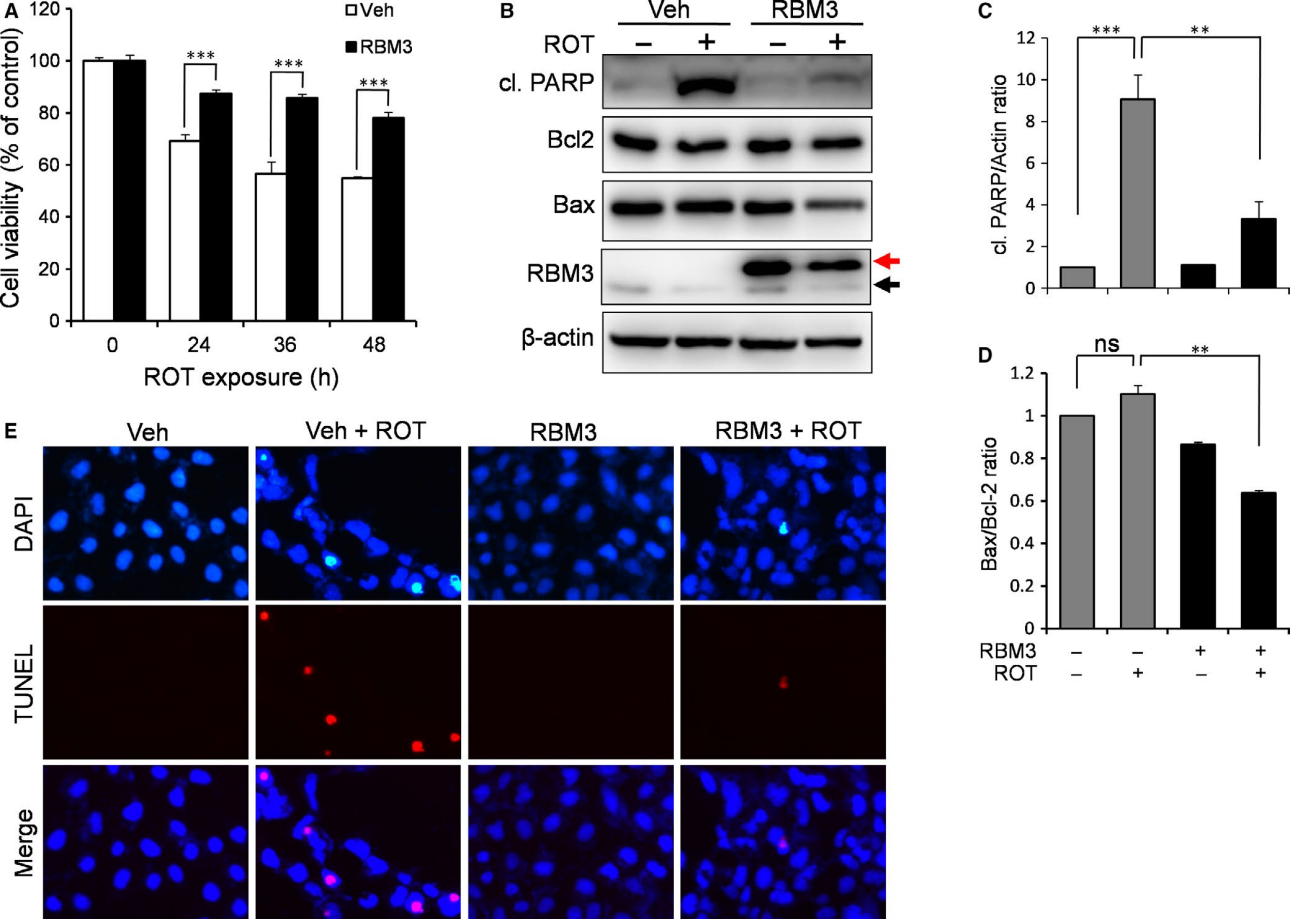
RBM3 protects against ROT‐induced apoptosis. SH‐SY5Y cells were transfected with the empty vector (Veh) or the RBM3‐expressing vector (RBM3) and treated with 0.5 μmol/L ROT. A, An MTT assay was performed to examine the effects of RBM3 overexpression ROT cytotoxicity after ROT treatment for a time course of 0, 24, 36 and 48 h. B, Cells were treated with ROT for 24 h before Western blotting for the indicated proteins. The black and red arrows indicate endogenous RBM3 and myc‐tagged RBM3, respectively. C, D, The levels of cl. PARP and Bcl‐2 were quantified by densitometry and normalized to β‐actin and Bax, respectively. E, TUNEL staining was performed after 24 h ROT exposure to evaluate the protective effects of RBM3 overexpression on ROT‐induced neurotoxicity. ***P < 0.001 vs vehicle‐transfected cells

We have shown above that RBM3 was strongly induced upon mild hypothermia treatment (Figure 1B). To evaluate the role of RBM3 in hypothermic neuroprotection against ROT, we overexpressed RBM3 in SH‐SY5Y cells by transfection with an RBM3‐overexpressing vector and assessed cell viability and apoptotic activity. The MTT assay showed a significant increase in cell viability at all time‐points of ROT treatment in cells overexpressing RBM3 compared to control (Figure 2A). Consistently, the levels of cleaved PARP were substantially reduced in RBM3‐overexpressing cells when compared to control cells transfected with empty vector (*P* = 0.0024; Figure 2B,C). Like the results seen after hypothermia exposure of ROT‐treated cells, the apoptosis‐related protein Bax, but not Bcl‐2, was down‐regulated in RBM3‐overexpressing cells (Figure 2B,D). TUNEL staining was lower in RBM3‐overexpressing cells compared to control cells, indicating that ROT‐induced apoptosis can be rescued by RMB3 (Figure 2E). Collectively, these data suggest that RBM3 protects SH‐SY5Y cells from ROT‐induced apoptosis.

### 3.3 RBM3 inhibits ROT‐induced activation of MAPK signalling

**Figure 3:**
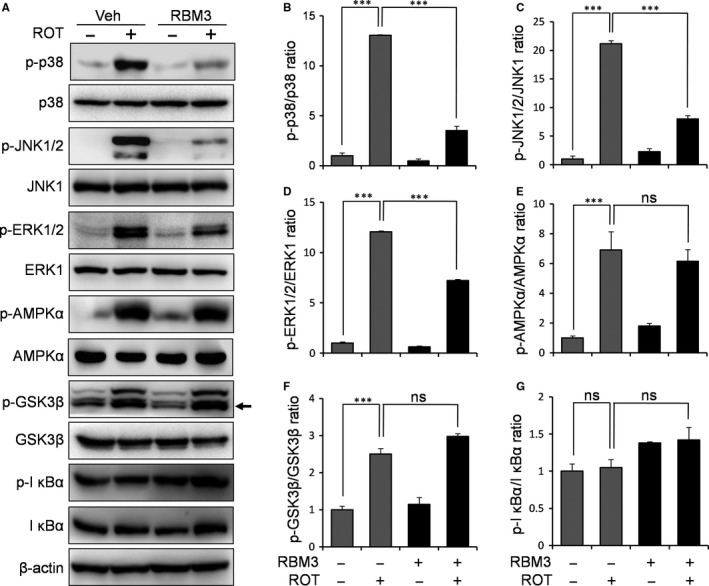
RBM3 inhibits ROT‐induced activation of MAPK pathways. SH‐SY5Y cells were transfected with the empty vector or the RBM3‐expressing vector and then treated with 0.5 μmol/L ROT for 4 h. A, Western blotting was performed to examine the levels of phosphorylated p38, JNK, ERK, AMPK, GSK‐3β and IκBα, with β‐actin as the loading control. The black arrow points to the quantified band for p‐GSK3β. B–G, The density of phosphorylated proteins was quantified and normalized to their respective total proteins. ***P < 0.001 vs vehicle‐transfected cells

To dissect the mechanism underlying the neuroprotective effects of RBM3 against ROT‐induced apoptosis, we studied the influence of RBM3 overexpression on ROT‐related signalling pathways. As shown in Figure 3A‐G, ROT treatment induced the activation of p38 (*P* = 6.5 × 10^−7^), JNK (*P* = 1.3 × 10^−8^), ERK (*P* = 0.000035), AMPK (*P* = 0.00063) and GSK‐3β (*P* = 0.00034) in SH‐SY5Y cells, but the NF‐κB pathway was not affected (*P* = 0.083). However, RBM3 overexpression inhibited ROT‐induced activation of all three MAPK signalling proteins (*P* = 0.000011, 1.1 × 10^−7^ and 0.00068 for p38, JNK and ERK, respectively; Figure 3A‐D), leaving AMPK, NF‐κB and GSK‐3β unaffected (*P* = 0.17, 0.056 and 0.26 for AMPK, NF‐κB and GSK‐3β, respectively; Figure 3E‐G). This indicates that RBM3 may prevent ROT‐induced neurotoxicity via inhibition of the p38, JNK and ERK pathways.

### 3.4 Mild hypothermia inhibits ROT‐induced activation of MAPK signalling

**Figure 4:**
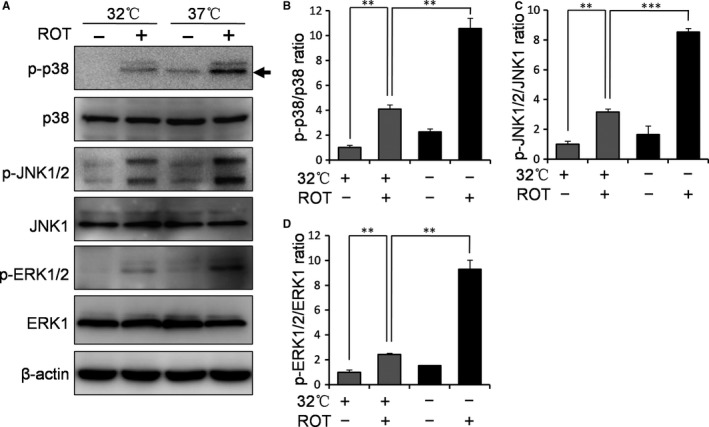
Mild hypothermia inhibits ROT‐induced activation of MAPK signalling in SH‐SY5Y cells. Cells were pre‐cultured under normothermic (37°C) or mild hypothermic (32°C) conditions for 1 d and treated with 0.5 μmol/L ROT for 4 h. A, Western blotting was then performed to evaluate the influence of hypothermia on ROT‐induced activation of p38, JNK and ERK pathways. β‐Actin was used as a loading control. The black arrow points to the quantified band for p‐p38. B‐D, The density of p‐p38, p‐JNK and p‐ERK was quantified and normalized to total p38, JNK and ERK1, respectively. **P < 0.01 and ***P < 0.001 vs normothermia pre‐cultured cells

As mentioned above, the overexpression of RBM3 attenuated ROT‐induced activation of MAPK signalling p38, JNK and ERK. Next, we tested whether mild hypothermia has the same effects on ROT‐induced activation of MAPKs. The Western blotting results showed that hypothermia markedly inhibits the activation of p38, JNK and ERK induced by ROT (*P* = 0.0071, 0.000012 and 0.0042, respectively, for p38, JNK and ERK activation; Figure 4A‐D), when compared with normothermic controls. These data suggest that both RBM3 and hypothermia inhibit the activation of MAPK signalling induced by ROT in SH‐SY5Y cells.

### 3.5 p38 signalling mediates the neurotoxicity induced by ROT in SH‐SY5Y cells

**Figure 5:**
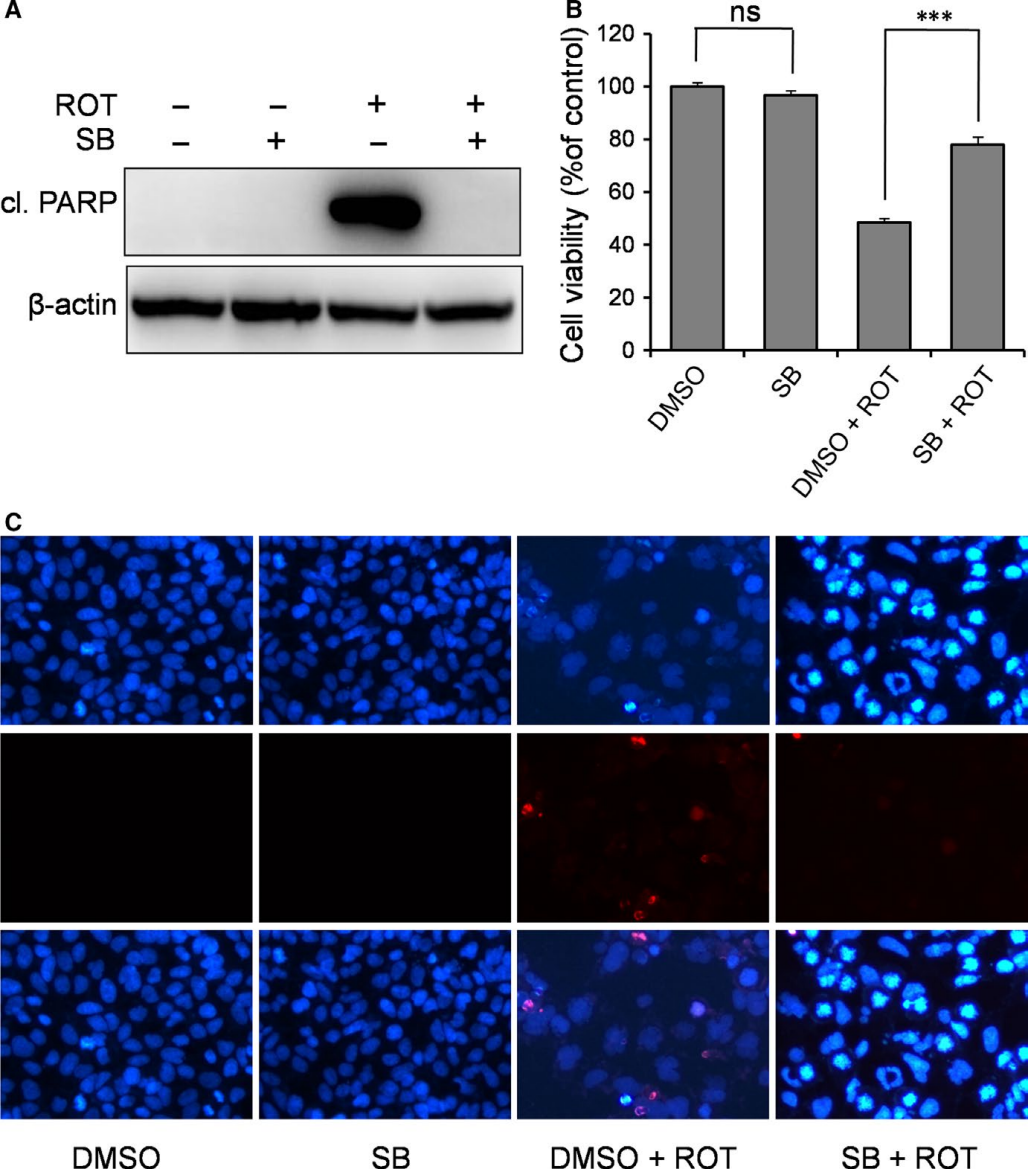
Inhibition of p38 signalling prevents ROT‐induced apoptosis in SH‐SY5Y cells. Cells were pre‐treated with p38 inhibitor SB203580 (SB, 10 μmol/L) for 1 h, followed by ROT (0.5 μmol/L) exposure for 1 d. A, Cleaved (cl.) PARP was detected by Western blotting, with β‐actin as the loading control. The MTT assay (B) and DAPI staining (C) were performed to evaluate the effect of p38 inhibition on ROT‐induced neurotoxicity. All nuclei are stained with DAPI (dark blue). ns: not significant, ***P < 0.001 vs DMSO‐treated control cells

As demonstrated above, either RBM3 or mild hypothermia inhibits ROT‐induced p38 signalling. We wanted to test if this blockade of p38 signalling is the mechanism behind RBM3’s neuroprotective effects in ROT‐treated cells. To this end, we used a p38‐specific inhibitor SB203580 to inactivate the pathway and measured the resulting effect on ROT‐induced apoptosis in SH‐SY5Y cells. As expected, blocking p38 caused a substantial decrease in the level of cleaved PARP (Figure 5A). Meanwhile, the MTT assay showed that the presence of SB203580 significantly reduced the cytotoxicity of ROT (Figure 5B), with cell survival rates increasing from 48.4% to 77.9% (*P* = 0.000099 for ROT vs ROT + SB). More convincingly, DAPI staining showed that apoptotic cell number was decreased in ROT‐treated SH‐SY5Y cells where p38 was inhibited (Figure 5C). Taken together, these data suggest that RBM3 prevents ROT‐induced apoptosis by inhibiting p38 signalling.

### 3.6 JNK signalling mediates the neurotoxicity induced by ROT

**Figure 6:**
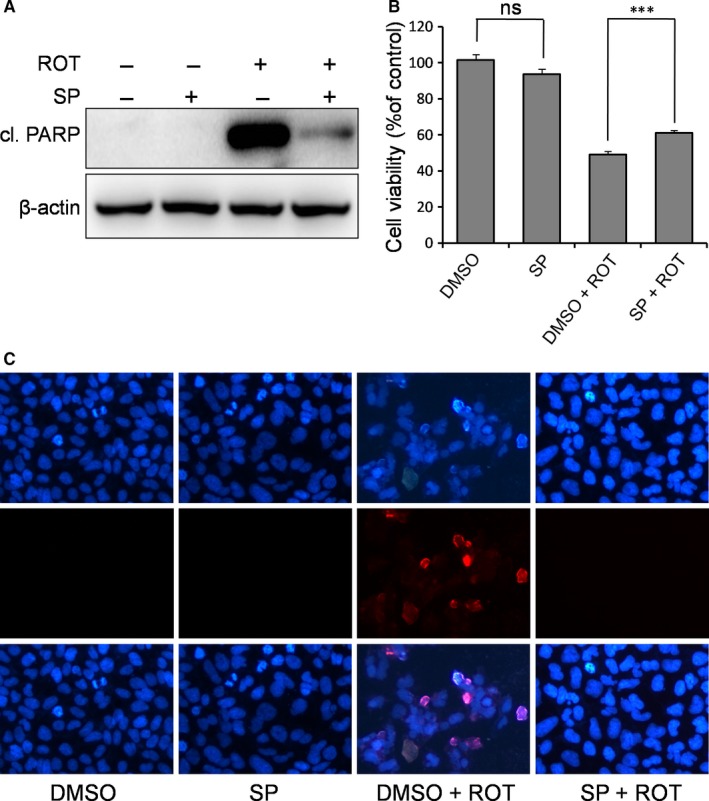
Inhibition of JNK signalling prevents ROT‐induced apoptosis in SH‐SY5Y cells. Cells were pre‐treated with JNK inhibitor SP600125 (SP, 25 μmol/L) for 1 h, followed by ROT (0.5 μmol/L) exposure for 1 d. A, Cl. PARP was detected by Western blotting, with β‐actin as the loading control. The MTT assay (B) and DAPI staining (C) were performed to evaluate the effect of JNK inhibition on ROT‐induced neurotoxicity. All nuclei are stained with DAPI (dark blue). ns, not significant, ***P < 0.001 vs DMSO‐treated control cells

As described above for p38, JNK activation by ROT was also inhibited by RBM3. To determine the role of JNK activation in ROT‐induced apoptosis, a JNK‐specific inhibitor (SP600125) was employed. In the presence of SP600125, the cleavage of PARP induced by ROT was almost completely abolished (Figure 6A). However, the presence of SP600125 only partially reduced the cytotoxicity of ROT as measured by the MTT assay (Figure 6B), with the survival rate increasing from 49.1% to 61.2% (*P* = 0.00061 for ROT vs ROT + SP). In addition, DAPI staining shows fewer apoptotic cells in the presence of the JNK inhibitor (Figure 6C). Collectively, these results indicate that RBM3 partially prevents ROT‐induced apoptosis via inhibition of JNK signalling.

### 3.7 ERK signalling mediates the neurotoxicity induced by ROT

**Figure 7:**
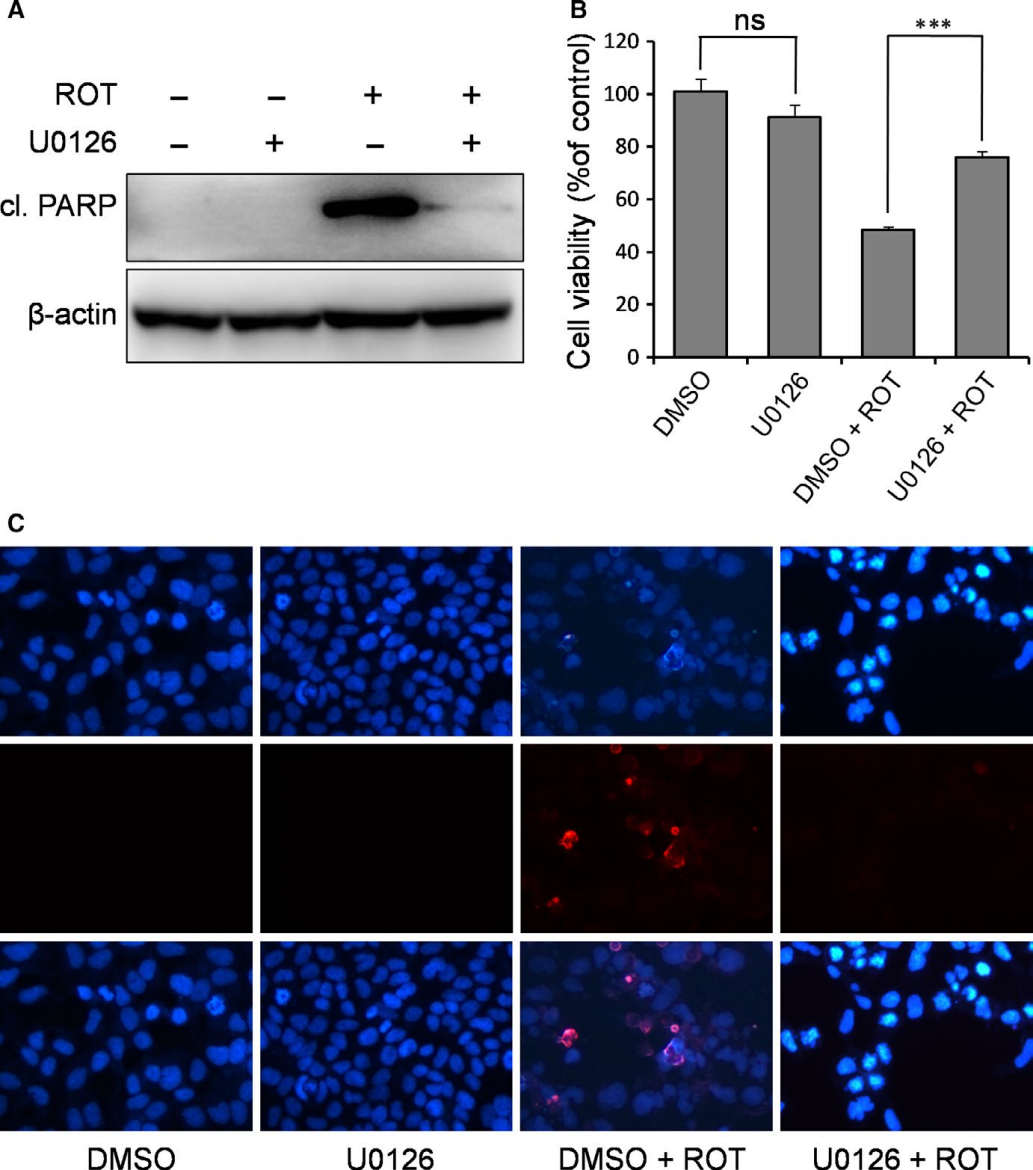
Inhibition of ERK signalling prevents ROT‐induced apoptosis in SH‐SY5Y cells. Cells were pre‐treated with MEK inhibitor U0126 (10 μmol/L) for 1 h, followed by ROT exposure (0.5 μmol/L) for 1 d. A, Cl. PARP was detected by Western blotting, with β‐actin as the loading control. The MTT assay (B) and DAPI staining (C) were performed to evaluate the effect of ERK inhibition on ROT‐induced neurotoxicity. All nuclei are stained with DAPI (dark blue). ns, not significant, ***P < 0.001 vs DMSO‐treated control cells

It is well known that ERK/MAPK functions as a pro‐survival signalling pathway in neuroprotection. Surprisingly, in the ROT‐based PD cell model, we observed the opposite; ERK was activated by the pro‐apoptotic ROT and was inhibited by the anti‐apoptotic RBM3. Therefore, in this experiment, we used a MEK‐specific inhibitor U0126 to directly examine the role of ERK pathway activation in ROT‐induced apoptosis. As shown in Figure 7A, in the presence of U0126, the cleavage of PARP induced by ROT was substantially diminished. Meanwhile, U0126 increased cell survival rate from 48.3% to 75.8% after ROT treatment (*P* = 0.000038 for ROT vs ROT + U0126; Figure 7B). Accordingly, DAPI staining showed a reduced apoptosis in cells treated with U0126, supporting that ERK inhibition prevents ROT‐induced apoptosis (Figure 7C). Together, these findings indirectly support the notion that RBM3 prevents apoptosis by inhibiting ERK signalling, and ERK signalling is pro‐apoptotic in ROT‐treated SH‐SY5Y cells.

## 4 DISCUSSION

PD is the second most common neurodegenerative disease and is characterized by a loss of dopaminergic neurons.^24^, ^25^ Some environmental risk factors, such as ROT and MPP^+^, are used as neurotoxins for evaluating potential neuroprotective strategies against apoptotic diseases like PD.^26^, ^27^, ^28^, ^29^, ^30^ For in vitro PD studies, the SH‐SY5Y human neuroblastoma cell line is frequently used as a cell model, because it exhibits many characteristics of dopaminergic neurons.^31^, ^32^ In the present study, we demonstrated that both RBM3 and mild hypothermia protect SH‐SY5Y cells from ROT‐induced neurotoxicity. Furthermore, our data also point out that RBM3 mediates mild hypothermia‐related neuroprotection against ROT by mainly inhibiting the MAPK signalling of p38 and ERK.

For the last century, hypothermia has been widely used as a clinical strategy used to alleviate the side‐effects of brain surgery and to provide neuroprotection after perinatal asphyxia.^11^ However, mild hypothermia is an aggressive clinical therapy, bearing side‐effects such as shivering, slurred speech or mumbling, slow and shallow breathing, low energy and weak pulse.^33^, ^34^ This inspired the search for molecular targets with fewer side‐effects to replace therapeutic cooling and led to the discovery of RBM3 as a crucial mediator of hypothermia‐related neuroprotection.^17^, ^18^, ^19^

Researchers have previously demonstrated that ROT induces neurotoxicity by caspase activation and apoptotic cell death, accompanied by the activation of pro‐apoptotic MAPK signalling pathways like p38 and JNK.^35^, ^36^, ^37^, ^38^, ^39^ Our study not only corroborated these results but additionally revealed that the neuroprotective effects of mild hypothermia in a ROT‐based PD cell model are mediated by RBM3. Importantly, we demonstrated that mild hypothermia prevents ROT‐induced neurotoxicity via induction of RBM3. Mechanistically, we found that RBM3 inhibited the ROT‐induced activation of p38, JNK and ERK signalling pathways. By using several different techniques to test the same predictions, we were able to strongly validate each result and increase the power of our data; PARP/caspase‐3 expression, the MTT assay and TUNEL staining examined apoptosis, and overexpression tools revealed the function of RBM3 in the PD cell model.

As opposed to p38 and JNK MAPK, the role of ERK activation in ROT‐mediated neurotoxicity is somewhat controversial. It was found that the histamine H2 receptor antagonist ranitidine prevents ROT‐induced neurotoxicity by activating ERK signalling,^40^ whereas neural growth factor prevents ROT‐induced neurotoxicity independent of ERK signalling.^36^ In this study, the ROT insult led to a strong activation of ERK signalling, and an ERK‐specific inhibitor clearly decreased the neurotoxicity induced by ROT, revealing a pro‐apoptotic role of ERK activation in response to ROT stimulation. The conflicting reports in the literature regarding the pro‐ and anti‐apoptotic roles of ERK1/2 are justified considering the diverse activation stimuli and downstream effectors in the ERK pathway that can alter the consequence of ERK1/2 on cell survival. Our observation is in line with findings by Zhao et al, which suggested that ERK activation is associated with the neurotoxicity elicited by ROT.^41^ Compared to the involvement of all three MAPK pathways in hypothermia/RBM3‐conferred protection against ROT neurotoxicity in this study, our previous studies found that inhibition of p38 signalling was the only MAPK pathway associated with neuroprotection by hypothermia/RBM3 against NO, RA and UV irradiation.^20^, ^21^, ^22^ These observations seem to indicate that p38 inhibition by hypothermia/RBM3 is a common neuroprotective mechanism in response to a variety of neurotoxins, while inhibition of JNK and ERK signalling is more specific.

Our finding that RBM3 rescues many of the deficits in the ROT‐based model of PD agrees with previous observations regarding the role of RBM3 in neuroprotection. In vitro, studies have demonstrated that RBM3 prevents apoptosis induced by excessive NO,^22^ UV irradiation,^21^ RA,^20^ staurosporine,^42^ hexanedione contact inhibition^25^ and serum deprivation,^43^ in cultured neurons or neuroblastoma cells. In vivo, it was recently found that RBM3 mediates the protective effects of hypothermia by reducing synaptic loss in a mouse model of Alzheimer's disease.^13^, ^44^ Therefore, we speculate that RBM3 induction in brains of PD patients may be a reasonable strategy for blocking PD progression caused by ROT. The trouble with RBM3 induction as a therapeutic strategy, however, lies in the extensive scope of its action. Altering cellular events like MAPK signalling can have very far‐reaching consequences whose cons may outweigh the pros. Further studies on the specific in vivo effects of RBM3 in nigrostriatal dopamine neurons affected by PD are needed to explore RBM3 induction as a viable alternative to hypothermia.

Interestingly, a recent study by us showed that mild hypothermia exacerbates MPP^+^‐induced apoptosis which was boosted when RBM3 was silenced by siRNA.^14^ Thus, mild hypothermia may have different effects on apoptosis in different PD cell models. However, a crucial product of hypothermia, RBM3, exerts same function on either MPP^+^‐ or ROT‐based PD models. In these two models, RBM3 silencing significantly boosts the apoptosis, whereas RBM3 overexpression markedly decreases the apoptosis. A possible reason is that RBM3 induction by hypothermia in MPP^+^ model is not enough to rescue apoptosis induced by MPP^+^ 14 In the future, MPTP‐ and ROT‐based PD mouse model should be used to determine the actual role of hypothermia in neurodegenerative diseases such as PD.

## CONFLICTS OF INTEREST

The authors report that there are no conflicts of interest.

## AUTHOR CONTRIBUTIONS

Mian Wang and Hai‐Jie Yang conceived and designed the research; Hai‐Jie Yang, Rui‐Juan Zhuang, Yuan‐Bo Li, Tian Li, Xin Yuan, Bing‐Bing Lei and Yun‐Fei Xie performed the experiments; Mian Wang and Hai‐Jie Yang wrote the manuscript; and Hai‐Jie Yang and Rui‐Juan Zhuang analysed the data.

## Data Availability

The data used to support the findings of this study are available from the corresponding author upon request.
