# Supplementary figures and images for: Cold‐inducible protein RBM3 mediates hypothermic neuroprotection against neurotoxin rotenone via inhibition on MAPK signalling
Source: J Cell Mol Med. 2019 Aug 22;23(10):7010–20. doi: 10.1111/jcmm.14588 (PMC6787511; doi:10.1111/jcmm.14588)

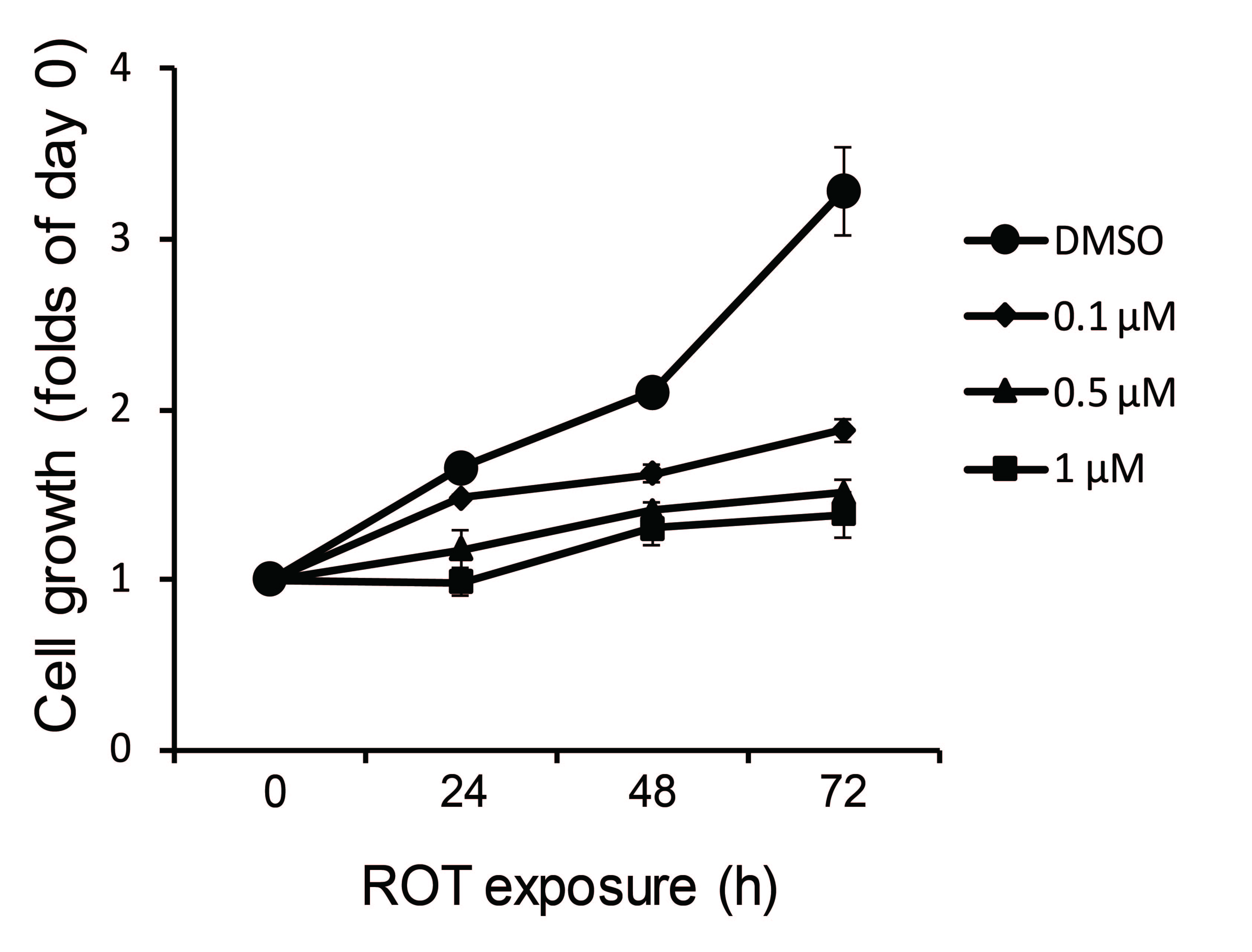

Supplement: Supplementary file 1 [file JCMM-23-7010-s001.jpg]
